# Multi-element lenslet array for efficient solar collection at extreme angles of incidence

**DOI:** 10.1038/s41598-020-65437-8

**Published:** 2020-05-26

**Authors:** Rakan E. Alsaigh, Ralf Bauer, Martin P. J. Lavery

**Affiliations:** 1 James Watt School of Engineering, University of Glasgow, Glasgow, UK; 2 Department of Electronic and Electrical Engineering, University of Strathclyde, Glasgow, UK

**Keywords:** Integrated optics, Solar energy and photovoltaic technology

## Abstract

Photovoltaics (PV) are a versatile and compact route to harness solar power. One critical challenge with current PV is preserving the optimal panel orientation angle with respect to the sun for efficient energy conversion. We experimentally demonstrate a bespoke multi-element lenslet array that allows for an increased power collection over a wide field of view by increasing the effective optical interaction length by up to 13 times specifically at large angles of incidence. This design can potentially be retrofitted onto already deployed amorphous silicon solar panels to yield an increased daily power generation by a factor of 1.36 for solar equivalent illumination. We 3D printed an optical proof of concept multi-element lenslet array to confirm an increase in power density for optical rays incident between 40 and 80 degrees. Our design indicates a novel optical approach that could potentially enable increased efficient solar collection in extreme operating conditions such as on the body of planes or the side of buildings.

## Introduction

Novel optical elements are an interesting and powerful route to address and resolve many engineering challenges. Recent advances in 3D printing technologies have revolutionised the manufacturing of such novel designs and enabled many solutions that cannot be achieved otherwise^1,2^. One area of interest is that of solar photovoltaics (PV)^3,4^, where the efficient collection of light over a large set of input angles is important. Unfortunately, only a fraction of solar energy can be efficiently collected by photovoltaic systems due to the physical constraints of optical back-reflections and optical interaction length (i.e. the effective optical path length within the PV absorber layer) over a wide range of acceptance angles. This limits the photon-to-electrical conversion in the absorbing layer within the semiconductor material^5–7^. The limited acceptance angle of PV is a key hindrance in its application for powering many mobile devices, autonomous drones and spacecrafts, as well as building-integration and domestic rooftops where complex tracking systems are not sustainable.

Traditionally, the angle-dependent efficiency constraints in PV have been tackled through the use of mechanical tracking systems that are expensive, require continual maintenance and an electrical monitoring system for autonomous operation. Static systems offer a clear advantage over mechanical systems in terms of maintenance, fault tolerance and installation. Many of the currently most promising researched passive optical solutions for this challenge require fundamental changes to industry manufacturing processes, which include light trapping techniques^8^, surface coating^9^ or surface integration of novel components^10^. However, given the maturity of PV production, substantial changes to manufacturing techniques are not ideal.

Optical coatings are commonly used in the fabrication of photovoltaic cells^11–14^. These cells are typically coated with multilayer antireflection (AR) materials to minimise the interfacial reflections of solar radiation for the entire solar spectrum^14^. Although advanced AR coating can substantially reduce optical losses for incident light, the performance of solar PV reduces significantly beyond 50°^5,6^. This is due to the increased angular reflection loss and short optical interaction length at extreme angles, which limit incident photons from effectively reaching the PV cells and hence reducing the effective electrical photovoltaic generation over a wide range of angles. Light trapping (i.e. the increase in the number of bounces of light within the PV absorber layer) is commonly used to increase photovoltaic generation by increasing the optical interaction length within the PV cell^15–24^. However, these solutions often have low efficiency at high angles of incidence^25–27^. Incorporating novel optical elements that can be added on-top of already manufactured solar PV surfaces to further promote light trapping over a wide field of view could significantly increase solar conversion efficiency (i.e. power conversion efficiency (PCE), which is the percentage of optical power that is converted into electrical power by the PV) at high angles of incidence.

**Figure 1:**
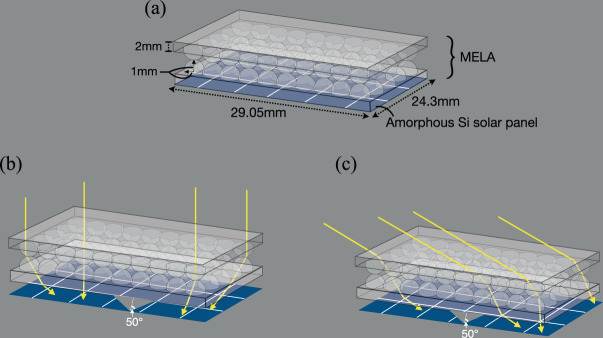
Ultra-wide angle multi-element lenslet array (MELA). (**a**) Schematic illustration of the system arrangement and dimensions, with the MELA integrated on-top of an amorphous Si PV panel. The MELA consists of two face-to-face layers of 2 mm diameter hemispheric lenslet arrays that sit on a 2 mm thick base. (**b**) Illustration of system with simulated rays entering the top lenslet array at an incident angle of 0° and spreading rays close to 50° to promote light trapping in the PV cell. The angle of the transmitted rays is largely dependent on the position at which the incident rays hit the top hemispheric lens. (**c**) Illustration of simulated rays entering the top layer at incident angle of 60° and transmitting through the opposite lens or collected by a neighbouring lens at angles close up to 50°.

**Figure 2:**
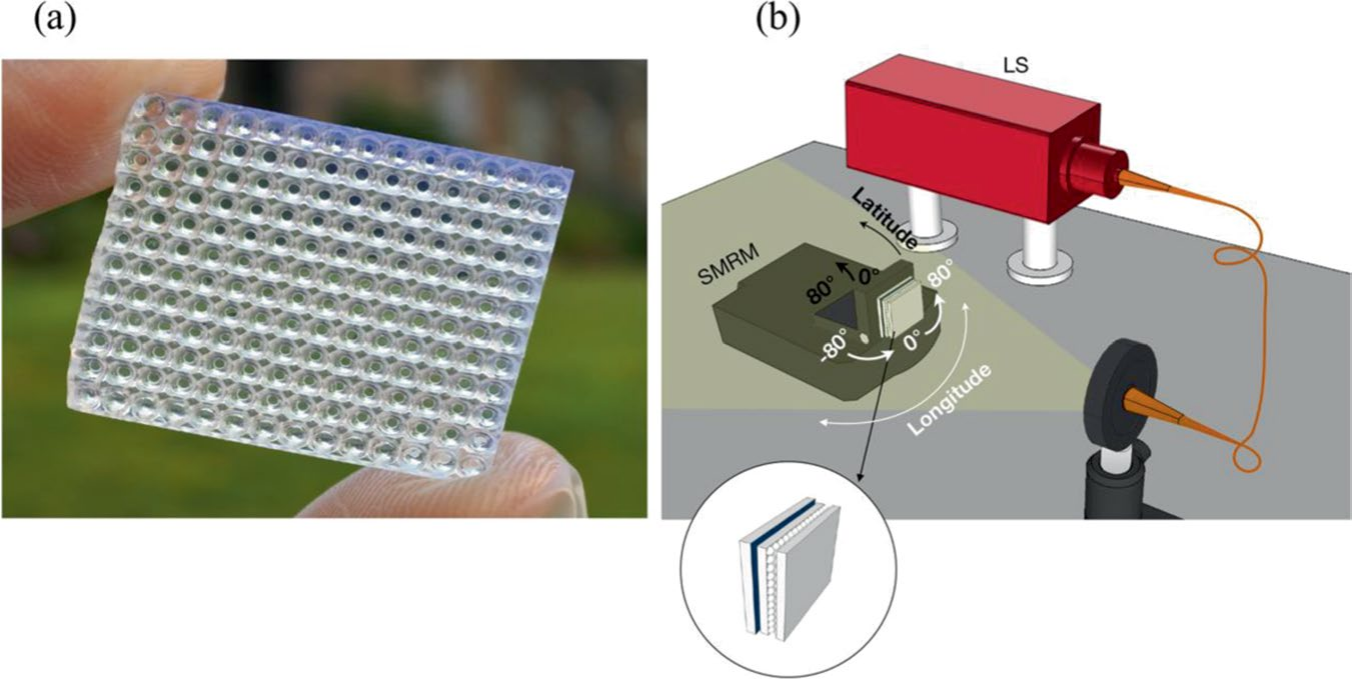
3D printed multi-element lenslet array (MELA). (**a**) Image of our 3D printed optical element. (**b**) Illustration of the experimental set-up, showing a fiber-coupled stabilised broadband light source (LS) illuminating a 18cm-diameter beam at the MELA’s plane. The optical element is on-top of an amorphous Si solar panel that is back-attached to a tilt-adjustable rotational mount driven by a stepper motor (SMRM) that provides the longitudinal and latitudinal angles.

In this paper, we outline the use of a novel multi-element lenslet array (MELA) that can be readily retrofitted onto solar PV surfaces to increase their solar conversion efficiency through the promotion of light trapping, specifically at high angle of incidence. The MELA comprises a grid of small aperture hemispherical lenses bonded with their curved faces touching, see Fig. 1, forming a simple easily manufacturable additional layer for PV panels. Rays entering one lens in the upper array layer are redirected and spread over a cluster of lenses in the second layer that subsequently redirects the rays within the solar cell to be close to angles that promote light trapping. Such angles are defined as the angles at which a ray would transmit to the PV absorber layer with minimum reflections and then achieve multiple bounces within that absorber layer through total internal reflection. The critical angle for these rays is equal or above 21°–37°, depending on the material, structure, encapsulation and coating of the device. The MELA increases the solar conversion efficiency by increasing the optical interaction length within the solar panel by redirecting rays to induce total internal reflections. System modelling shows that the MELA design increases the total optical interaction length within the panel by a factor of 13 times over wavelengths of 450–1050 nm, which leads to an increase in power collection of 1.36 over incident angles ranging from 0°–80° that replicates a full day of solar collection. Experimental tests were performed with a 3D printed prototype lenslet array, see Fig. 2, bonded to a commercially available solar panel (Sanyo, AM-1815CA), which can be readily retrofitted to new or already deployed solar panels. The experimental MELA increased power generated by the PV cell by a factor of 1.038 over a full day at equatorial latitudes. Rays incident at the lenslet array with angles of 80° are collected with an enhancement factor of between 50 and 250 depending on latitude. This potentially allows solar collection to be used in extreme operating condition, where MELA increases the conversation efficiency from 4.6% to over 8% at angels of incidence beyond 50°. We expect these increases to potentially allow more efficient use of solar PV in extreme operating conditions such as on the body of planes or the side of buildings.

## Results

### Optical element design

We take a novel approach to the design of light trapping optical structures by implementing a multilayer hemispheric lenslet array to maintain efficient optical performance for normally incident rays, yet promote increased efficiency for light incident at high angles of incidence. An ideal optical system for efficient solar collection would redirect input rays from any arbitrary input angle to a single propagation direction that leads to optimal energy conversion within the photovoltaic panel. However, conservation of étendue prevents this from happening^28^. Combing two hemispheric lens, with their curved faces touching, will produce wide field of view optical systems such as those proposed by Rezaei *et al*.^29^ Such optical structures have not been used in combination with solar PV for the collection of rays from a large set of input directions. In our optical system, two lenslet array layers, where each lenslet has an aperture size (2 mm) and short focal length (2 mm), are positioned and optically bonded with their curved faces touching, as shown in Fig. 1. These lenslet arrays are placed shorter than one focal length away from each other, which means after propagation through both layers, normally incident rays are spread over a wide angle in a similar manner to defocus from a misaligned imaging system. Additionally, light illuminating the system at high incidence angles are spread due to deflections that occur by the off-center illumination of a lens, Fig. 1(c). Each of these forms of optical deflection promote light trapping within the PV and lead to an increased conversion efficiency for light illuminating the optical system.

**Figure 3:**
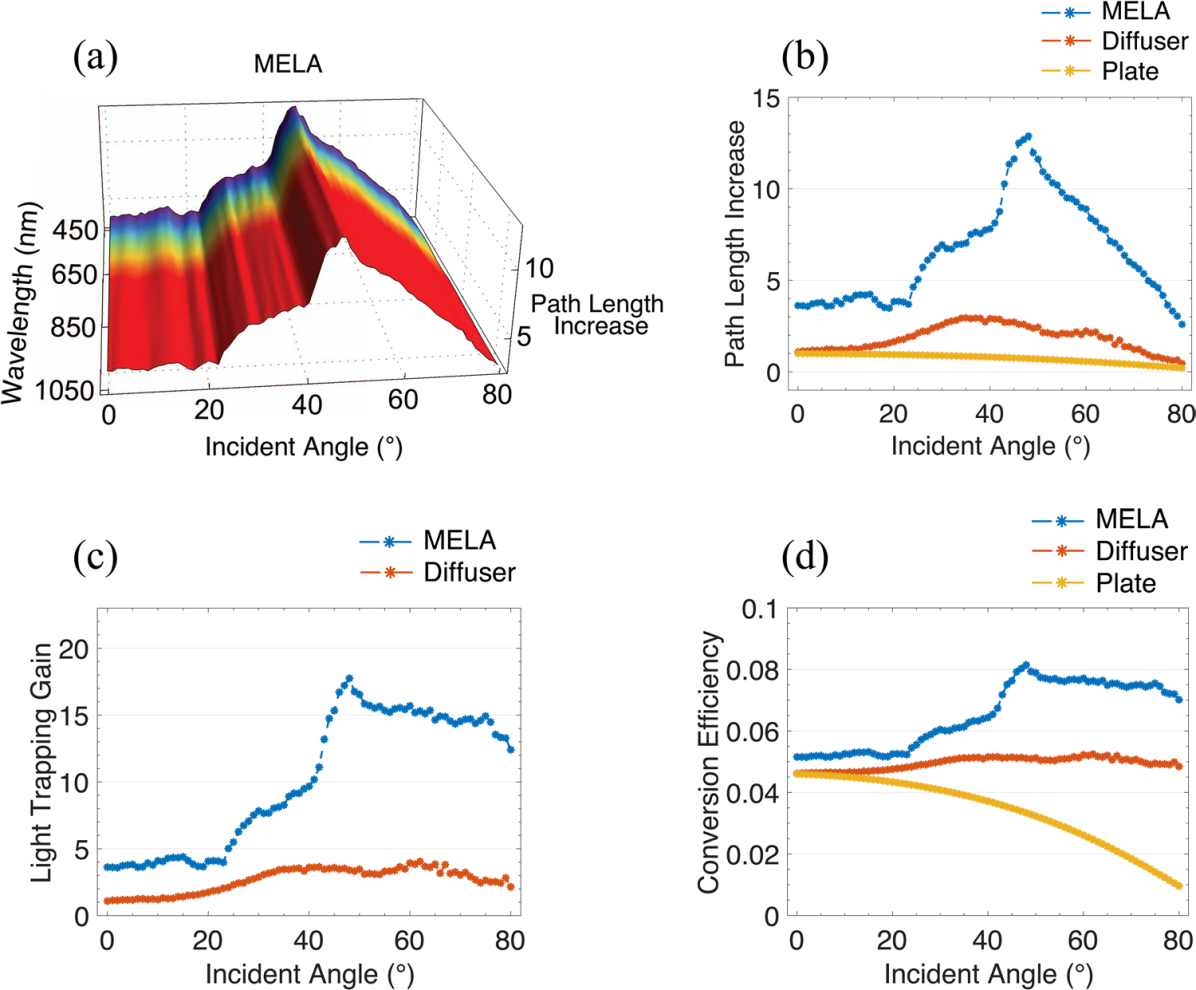
Path length and light trapping gains. (**a**) Optical path length increase for rays transmitted through our MELA as a function of both wavelength and incident angle, yielding a consistent performance along different wavelengths. (**b**) The increase in optical path length is simulated for rays that would reach a PV cell after being transmitted through a flat plate, diffuser plate and compared to our MELA. Our MELA can significantly increase the path length of rays incident at high angles, and outperforms a flat plate and randomly textured diffuser plate for all incident angles. This increase in path length is due to the ability of our MELA to spread incident rays from a wide range of input angle in a controlled manner that is within the PV acceptance window and effectively prolongs their optical interaction length. (**c**) Gain factors in the trapping of light for our MELA and diffuser plate over a flat plate, where the plate is used here as the normalising factor. (**d**) Efficiency gains with angle arising from the promotion of light trapping in an a-Si solar panel with a standard efficiency of 4.46%.

Using Comsol Multiphysics, we modelled the increase in optical light trapping arising from the expected addition of the MELA, see Fig. 3. This model employed our MELA design on top of a PV absorber layer with 1 mm depth. A 1000 W/m^2^ illumination was released into the total input aperture of the MELA and the angles of the rays reaching the back surface of the absorber layer with sufficient optical power were computed. The expected path lengths of each ray were then derived through geometrical analysis of the angle of incidence and ray position to fully assess the trapping of these rays within the PV cell. The accumulative path length was then computed for wavelengths between 450–1050 nm, sampled at steps of 50 nm, and over incident angles of 0°–80° in a step of 1° for rays with a power density equivalent to or greater than 4.39 mW/m^2^, as shown in Fig. 3(a). This power threshold was calculated by determining the minimum power per unit mm required for successful electrical conversion in a standard amorphous Silicon solar cell with a band gap energy of 1.8 eV, doping density of 1 × 10^20^ per cm^3^ and maximum light trapping gain of up to 50^30^. Upon analysing a wide range of commercially available amorphous silicon panels, we used a simulated panel with expected power conversion efficiency of 4.46%, see Table S1 in supplementary material. In this simulation, we assume the material of the MELA to be Zeonex E48R polymer, as this polymer has similar refractive indices as the 3D printing material used in our experiments (n = 1.5403)^31^. Our calculations also assume a constant attenuation of the signal along and within the PV, and do not consider any rays that are below the band gap energy. We note that such path length calculations could change depending on the PV internal structure. However, this is variable between commercial panels and assuming an optical guiding to be the best approximation for our particular calculation.

Further, we directly compare our MELA to a 1 mm flat optical layer and randomly textured diffuser surface that is widely reported structure to achieve light trapping in solar cells^17,21,32^, where the refractive index used is also equivalent to our 3D printing material. The expected accumulative path length within the PV cell is calculated for the MELA, flat plate and diffuser plate, shown in Fig. 3(b). We also compute the light trapping of the MELA and the diffuser plate with respect to the flat plate, as shown in Fig. 3(c). These models assume that the PV surface is bonded to the transmitting surface of the elements, and the absorber layer is 1 mm within the PV. These results confirmed that our MELA increases the path length of rays at all incident angles with significant increases for angles above 30°, leading to an efficient conversion at extreme angles. Computing the expected power increase over a full day of operation with a conversion efficiency of 4.46%, model predicts the MELA will provide a multiplicative gain factor for 1.361 for a PV panel with 4 PV cells with an area of 211.7 mm^2^. The simulated diffuser would lead to a gain factor of 1.069. These increased path length and light trapping are expected to yield a gain in conversion efficiency of over 8% at high angles using our MELA for an amorphous Si solar panel with a standard efficiency of 4.46%, as shown in Fig. 3(d). It should be noted that such gains are for amorphous silicon and could vary based on the specific efficiency of the panel being used. These simulations show a considerable increase in path length specifically at angles above 30°, hence increasing the performance of solar collection at extreme angles of incidence towards the sun.

**Figure 4:**
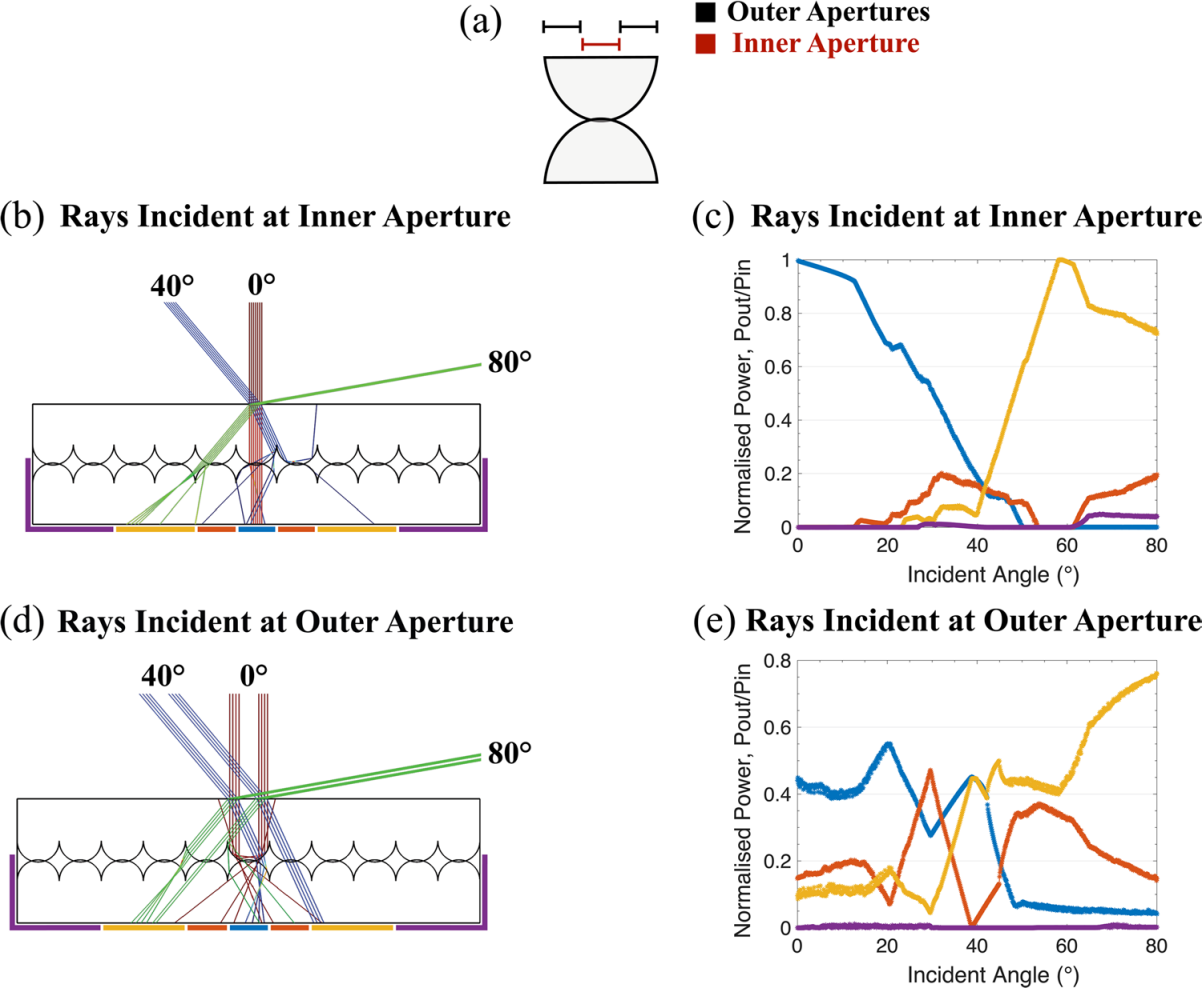
A simplified ray diagram, with a reduced illumination array, to illustrate the optical propagation through the multilayer lenslet. (**a**) Illustration defining two distinct illumination areas used in this simplified ray diagram. (**b**) Illustration of a ray-traced simulation for rays entering a 0.67 mm region at the centre of the aperture (inner) of a single lenslet on the top optical surface for incident angles of 0°, 40° and 80°. This area approximately corresponds to physically bonded area joining the two lenslet elements, therefore ray that are incident at small angles are predominately propagate undeflected. Rays at higher incident angles, 40° and 80° here, are distributed over a larger area leading to increased path length (light trapping) in the bonded solar cell. (**d**) Light entering an outer annulus of the single lenslet with an inner radius of 0.67 mm and an outer radius of 1.33 mm, shows considerable deflection of the rays. (**b**,**d**) The power arriving at specific regions can be modelled for angle between 0 and 8°0 in 1° steps.

The two-layer multi-element construction of the lenslet array allows for rays entering one lens on the top layer to be collected by a cluster of lenses in the second layer. To specifically map the ray propagation, we limit the area that rays are incident to a simplified version of our optical system. With this system, we consider one single lenslet in the top layer and separate this into two specific areas of the aperture, the central 1/3^rd^ of the lenslet (inner aperture) and an outer annulus that covers the remainder of the lens aperture (outer apertures), Fig. 4(a,b,d). These results illustrate an increase in the illuminated area of the active PV surface as light propagates from one layer to the next, Fig. 4(b,d). The spread can be calculated by measuring the power in defined regions at the bottom surface of the MELA, Fig. 4(c,e). The angular spread after the second lenslet means many of the rays will now be totally internally reflected and will be trapped, hence increasing the effective path length within the PV. Our simulation shows that the average angle of all the rays transmitting through our MELA is 29.4° over incident angles of 0°–80° with a standard deviation of 19.5°, in contrast to average angle of only 18.6° with a standard deviation of 11.4° for a flat plate. Beyond light trapping, diffusion of optical rays has also been previously reported to minimise losses from PV internal electrical contacts and minimise local saturation effects within the panel, as detailed by the refs. ^33–35^, therefore potentially further increasing conversion efficiency.

### Prototype fabrication

**Figure 5:**
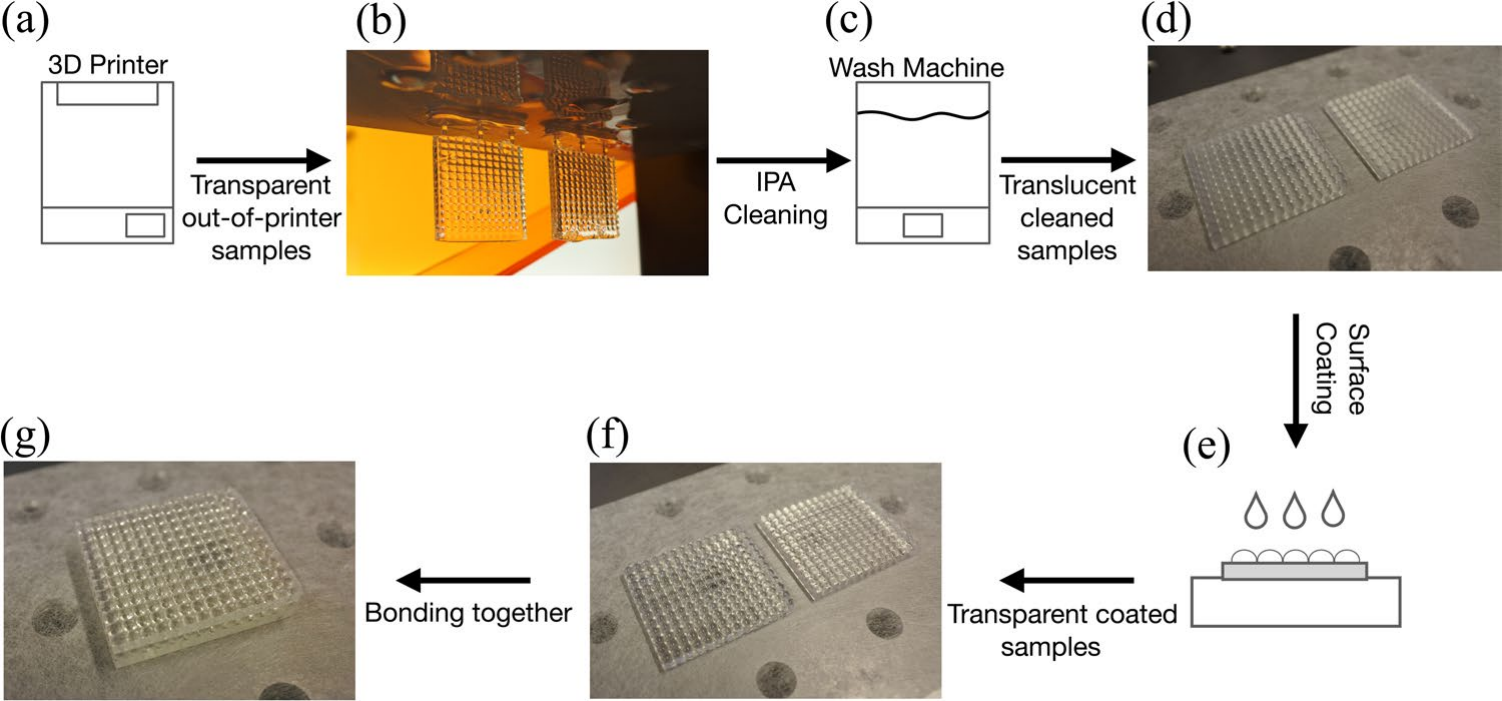
3D printed optical element post-processing procedures. (**a**) Stereolithographic 3D printer to fabricate the lenslet array. (**b**) Image of out-of-printer lenslet arrays looks transparent due to accumulated resin on the surface. (**c**) Wash machine filled with Isopropyl alcohol (IPA) to clean the samples. (**d**) Image of cleaned lenslet arrays as they look translucent due to surface roughness that was previously covered by resin before the cleaning. (**e**) Surface coating with index-matched material to smoothen the surface and makes it transparent. (**f**) Post-coating image of transparent lenslet arrays. (**g**) Lenslet arrays are tip-bonded together to form the optical element. The alignment of the two arrays was performed through mechanical alignment of the four corners of the two bases that the lenses sit on to guarantee the accuracy of the lenses, without particularly stringent alignment requirements. This is due to both arrays having the same shape and size, and dimensions. We measured the tolerance of our alignment to be 60 µm and our simulated system confirms a minimal impact on performance for up to 100 µm in misalignment.

Due to the novel nature of the optical lenslet array, the prototype lens was manufactured through the use of micron-resolution high-precision stereolithographic 3D printing. Recent advances in 3D printer technology have enabled the formation of clear photo-polymerised samples, which have been demonstrated in various applications^36–39^. The printing of these materials combined with specific post-processing procedures has improved the surfaces roughness to the nanometer scale^1,36^. We produced optical quality lenslet arrays using a widely available 3D printer combined with several stages of post-processing, see Fig. 5. Each of our hemispheric lenslet arrays was printed separately and subsequently bonded with medium-viscosity UV-curable index-matched fluid, providing a transparent boundary layer joining the curved faces, see Fig. 1. The 29.05 × 24.3 mm optical element was then directly fitted to a standard commercial amorphous silicon solar panel using index-matching fluid to minimise intermediate reflection losses of light at the boundary between our lenslet array and the solar panel. The active area of this panel was also size-matched to be 29.05 × 24.3 mm with a light absorbing tape to be equivalent to the optical element aperture. Further, we tested the heat resilience of these plastics in direct solar illumination at ambient temperatures of over 50° Celsius and observed no noticeable damage to the 3D printed materials.

The transmission loss associated with the 3D prints was determined by using the broadband white light source. Using a spectrometer, the overall optical loss for our MELA was measured to be 20.7%. To determine the contribution for absorptive losses versus back reflective losses, we measured the loss from flat surfaced 3D printed boxes of varying thicknesses, as detailed in the supplementary material. Based on these experimental measurements, we calculated the plastic absorptive loss to be approximately 1.3% per mm, which likely occurs due to the internal plastic layering, and reflective losses per optical surface of approximately 4.3%, see supplementary material. Our measured loss shows a slight improvement compared to previous research that employs similar approaches to 3D printing optics^37,40^.

Figure 5 illustrates the steps taken to 3D print and post-process the lenslet arrays. A stereolithography (SLA) 3D printer was used to fabricate the lenslet array from a liquid resin using a λ = 405 nm high power laser in a layer-by-layer photopolymerisation process at a layer thickness of 50 μm. Due to the nature of SLA printing that forms solid elements from liquid material, the lenslet arrays were cleaned in 90% isopropyl alcohol (IPA) to remove accumulated uncured resin and other residues from the surface. The clean surface was coated with UV-curable index-matching material to smoothen the surface and enhance its transparency. The lenslet arrays were tip-bonded and cured using a λ = 405 nm UV light to form the optical element.

### Experimental performance

To experimentally investigate the feasibility of our prototype design, we measured the short circuit current density (J_sc_) and the open circuit voltage (V_oc_) across an “off-the-shelf” amorphous silicon solar panel (Sanyo, AM-1815CA), over a wide range of illumination angles using a fiber-coupled stabilised white light source as solar model. This particular cell was selected due to its lack of internal light trapping structures, allowing us to confirm the behaviour of our novel light trapping layer. As this is a commercial solar panel, calculation of the theoretical power conversion efficiency could not be determined directly from the manufactures datasheet. To remove the requirement for an accurate determination of the panel efficiency calculation, all our presented data of short circuit current densities and open circuit voltages using the MELA are compared to experimentally measured baseline values for voltage and current. The same illumination source was used for the collection of all experimental results. Utilising a stepper motor-driven rotation mount, a range of solar illumination angles were tested over the range −80° to 80° in the longitudinal axis, where a further mechanical rotation mount was used to allow for tilt of the solar panel for latitudinal alignment. We used a 400 µm core optical fiber (Thorlabs, M28L01) with around 19.5 cm propagation distance to the solar panel, yielding a uniform illumination with around 18 cm beam waist at the solar panel plane that covers the full collection aperture and mimics solar radiation, as shown in Fig. 2(b). Further, we tested the beam focusing of our illuminating beam using a plano-convex lens at the solar panel plane and observed a spot that is consistent with the focusing of the solar illumination.

**Figure 6:**
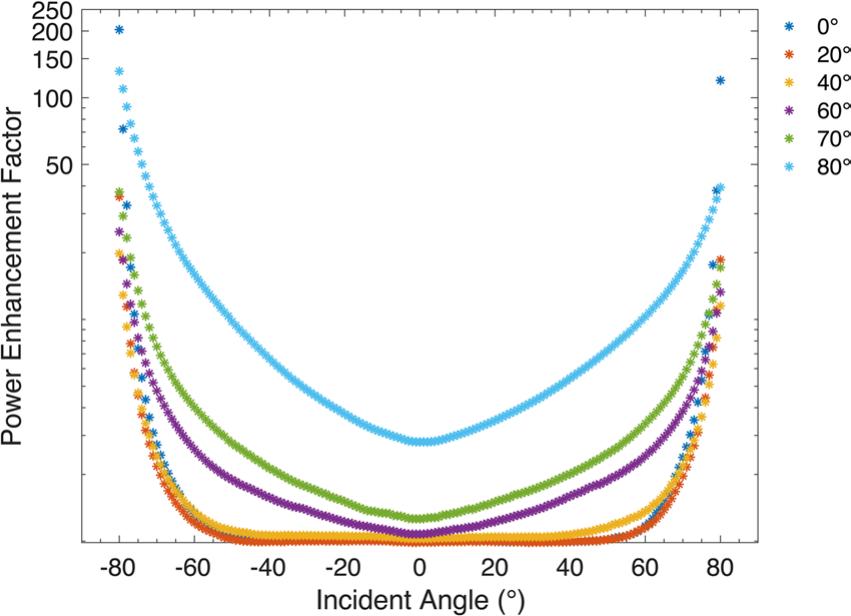
Experimental power increase factors for various tilt angles. Multiplicative increase factors in the power density produced by an off-the-shelf amorphous silicon solar panel (Sanyo, AM-1815CA) for the addition of our 3D printed MELA over the flat panel, at panel latitudinal tilt angles of 0°, 20°, 40°, 60°, 70° and 80°, as a function of incident longitudinal angles ranging from ±80°. Due to the variation in the lower power threshold of different solar panels, these gains are dependent on the product of the J_sc_ and V_oc_ and might vary depending on the solar panel itself.

Our white light source (Thorlabs, SLS201L) provides a black body radiation with an optical power that leads to panel saturation, at 40 W/m^2^ at a temperature of 2796 K. We fully characterised the performance of the solar panel using our light source prior to the MELA integration, where a part of each side of the panel was covered with light absorbing tape at the boundary between cells to fit with the size constraints in our manufacturing process and prevent unintended bleed of light into any adjacent regions. This reduction altered the active area to 705.9 mm^2^ from 2535.7 mm^2^ to match the size of our 3D printed MELA. The device characterisation was done over the full range of possible angles of incidence, where J_sc_ and V_oc_ measurements were taken in 1° of longitudinal steps at a fixed latitude tilt of 0° with a measurement time of 2 seconds at each step, see Supplementary Fig. S1. Based on these measurements the power density produced by the panel was also calculated, see Supplementary Fig. S1. The fluctuations in the measured current and voltage were found to be small with a cumulative standard deviation between the samples of 2.134 nA and 6.937 mV, respectively. Subsequently, a measurement over the identical longitudinal sweep is taken using the same panel with our lenslet array bonded to the surface, see Supplementary Fig. S1. The multiplicative gain factors measured in the power density for the addition of our lenslet array over the flat solar panel are shown in Fig. 6. It is important to note that these experimental increases were measured for a commercial amorphous Si solar panel under standard conditions and could change depending on various factors, including panel technology, fill factor and temperature of the panel that could affect the overall performance of the device^41^.

The daily changes in sun position are only one of the issues that limit the efficiency of solar technologies. Over the course of the year the suns latitude in the sky changes, leading to a secondary angle dependent efficiency change. Even more importantly, any mobile platforms that intend to use solar power (planes, drones, etc.) will never have a guaranteed solar alignment. To test the applicability of our system for such applications we altered the effective latitude by varying the adjustable mount angle with respect to the incoming light. For a selection of five fixed latitude angles, a further scan over the longitudinal range discussed earlier was taken, where the J_sc_ and V_oc_ were measured and the power density was calculated prior and post MELA integration to the PV panel, see Supplementary Figs. S2–S6. The power increases for the addition of the MELA are shown in Fig. 6. We measured an increase factor in full day power of 1.038 ± 0.002, 1.028 ± 0.002, 1.097 ± 0.001, 1.394 ± 0.001, 1.768 ± 0.001 and 4.783 ± 0.002 at latitude angles of 0°, 20°, 40°, 60°, 70° and 80°, respectively. We note that these electrical power gains are functions of both the J_sc_ and V_oc_, which vary linearly and logarithmically with the optical intensity respectively depending on the solar panel material, illumination levels and conversion efficiency^42^. A summary comparison of cumulative experimental values of Jsc, Voc, power density and power enhancement factor for a range of latitude input angles is shown in Table 1.

**Table 1:** Cumulative experimental measurements of short circuit current density (J_sc_), open circuit voltage (V_oc_) and power density for the amorphous silicon solar panel (Bold) and with the addition of the MELA (italic) over longitudinal angles of ±80° for latitudinal angles of 0°, 20°, 40°, 60°, 70° and 80°. Based on the cumulative power density, the power increase factor is calculated for the addition of the MELA over the flat panel.

Latitude Angle (°)	J_sc_ (μA/cm^2^)	V_oc_ (V)	Power Density (μW/cm^2^)	Power increase factor
0°	**4.078**	**136.45**	**4.104**	1.038 ± 0.002
	*4.279*	*143.26*	*4.262*	
20°	**4.043**	**135.89**	**3.985**	1.028 ± 0.002
	*4.206*	*141.21*	*4.098*	
40°	**3.734**	**127.15**	**3.457**	1.097 ± 0.001
	*4.047*	*136.31*	*3.794*	
60°	**2.693**	**94.89**	**1.945**	1.394 ± 0.001
	*3.388*	*117.07*	*2.713*	
70°	**1.781**	**64.67**	**0.880**	1.768 ± 0.001
	*2.487*	*93.31*	*1.556*	
80°	**0.762**	**27.81**	**0.160**	4.783 ± 0.002
	*1.793*	*66.55*	*0.769*	

The power enhancement factors presented in Fig. 6 show increases in the overall power produced by the panel at angles above 50°, with insignificant losses at normal incidence, when our 3D printed MELA is bonded to the solar panel. Even though our prototype MELA has shown an optical loss, this loss does not map directly into electrical power loss due to the previously described current and voltage dependencies on optical intensity. The proportionality between the optical power and the power density produced by our PV panel was experimentally characterised using neutral density (ND) filters, see Supplementary Fig. S7. At our experimental power levels this characterisation indicates that the reduction in the optical power by 20.57% results in a 12.23% loss in the measured electrical power. From the simulation, it is expected that the addition of our MELA yields a 5 times gain in light trapping and increases the optical efficiency by 23% (i.e. the percentage of optical power absorbed by the PV). These increases are expected to mitigate the optical losses that might occur when our element is used at low angles of incidence. When considering losses and the power performance of our a-Si solar panel, our simulation would lead to a full day power increases factor of 1.086, where we expect the difference in full day performance is arising from the surface roughness of the 3D printed optical element. One could mitigate these losses through the use of anti-reflection coated optical surfaces and use of a higher precision 3D printer or injection moulded plastics.

## Conclusion

We have developed a novel multi-element lenslet array (MELA) that can be used to increase efficiency of solar collection at angles beyond those of traditional optical systems. This MELA employs controlled scattering to provide an increase in the usable optical power incident on the solar panel, increase the effective optical interaction length and promote light trapping. Balancing each of these features in a lenslet array form provides a novel method for designing non-imaging optical systems for optical collection. The optical improvements achieved through the combination of these features were demonstrated in our simulation model, and experimentally confirmed with electrical measurements using an a-Si solar panel. Our simulation models have shown an increase in optical path length and light trapping by bonding our MELA onto a PV cell. Since these increases are directly proportional to the short circuit current density produced by the panel^35^, the gains in our experimental measurements are due to the higher light absorption achieved by the trapping of light. The slight difference between the simulated gain and actual measured performance is expected to be arising from the losses of the prototype 3D prints. Although our light trapping approach does not outperform the world-record holding devices that use integrated textured pyramid structures^43,44^, our approach introduces a very compelling route for additive layer light trapping technologies that could be used to upgrade older technologies or potentially provide alternative routes to manufacturing panels containing light trapping technologies. Furthermore, we expect this principle could be optimised for further increases in conversion efficiency. Lenslet arrays that can efficiently collect light over a large range of incidence angels could be used for aerial vehicles that have no fixed angular relationship with the sun, such as planes and permanently airborne drones like those used for enabling future generation telecommunication networks^45^. Given the hemispherical nature of the lenslet array design, industrial processes such as injection moulding could be used to allow for mass production of this prototype.

## Supplementary information

Supplementary information.
