# Supplementary material for: Multi-element lenslet array for efficient solar collection at extreme angles of incidence
Source: Sci Rep. 2020 May 26;10:8741. doi: 10.1038/s41598-020-65437-8 (PMC7250910; doi:10.1038/s41598-020-65437-8)
Supplement: Supplementary file 1 — Supplementary information. [file 41598_2020_65437_MOESM1_ESM.docx]

Supplementary Information for

Multi-element lenslet array for efficient solar collection at extreme angles of incidence

Rakan E. Alsaigh,^1,3^ Ralf Bauer,^2^ AND Martin P.J. Lavery^1,4^

^1^School of Engineering, University of Glasgow, Glasgow, UK

^2^Department of Electronic and Electrical Engineering, University of Strathclyde, Glasgow, UK

^3^r.alsaigh.1@research.gla.ac.uk

^4^martin.lavery@glasgow.ac.uk

**Transmission and absorptive losses of the 3D prints**

The transmission and absorptive losses of our 3D prints were experimentally evaluated using a broadband white light source (Thorlabs, SLS201L) and a spectrometer (Thorlabs, CCS200/M) and flat surfaced 3D printed bulks of material (boxes) with a varying thickness. The propagation distance between the light source the spectrometer was 19.5 cm to match the propagation distance used between the source and the solar PV in our experiment, with the 3D prints placed 1 cm away from the spectrometer. The thicknesses of the 3D prints were varied between 1 mm to 6 mm (total thickness of our element) to assess the absorption of the material as a function of thickness. The drop in light intensity was measured using the spectrometer with and without these 3D prints to be between 10.1% and 16.4%. To further look at the absorption of a bulk that is thicker than our element, an intensity drop of 20.5% was also measured for an 8 mm flat surfaced 3D printed box. Using these losses, we computed the bulk absorptive loss as

$$Absorption per surface=\frac{\% Loss of the 8 mm box-\% Loss of 1 mm box}{Thickness of the 8 mm box}= \frac{20.5\%-10.1\%}{8}=1.3\%$$

Using this absorptive loss, the back reflective loss was then calculated as

$$Reflection per surface=\frac{Total loss-Absorption}{Number of surfaces}= \frac{16.4\%-(1.3\%*6)}{2}=4.3\%$$

Noting that the total loss and the absorption here were taken for the box with the equivalent thickness as our MELA, i.e. 6 mm.

**Analysis of standard efficiency of commercial amorphous silicon panels**

| **Manufacturer** | **Part No.** | **Vop (V)** | **Iop (A)** | **Area (m^2^)** |  | **Efficiency (%)** |  | **Datasheet links** |
| --- | --- | --- | --- | --- | --- | --- | --- | --- |
| Panasonic - BSG | AM-5302 | 1.7 | 0.105 | 0.00367536 |  | 4.8566671 |  | <https://docs.rs-online.com/2167/0900766b80d10ce8.pdf> |
| Panasonic - BSG | AM-5308 | 1.7 | 0.0688 | 0.00236472 |  | 4.94604012 |  | <http://www.farnell.com/datasheets/87125.pdf> |
| Panasonic - BSG | AM-5412 | 2.2 | 0.0398 | 0.00165831 |  | 5.28007429 |  | <https://docs.rs-online.com/dd86/0900766b80d10cf7.pdf> |
| Panasonic - BSG | AM-5413 | 2.2 | 0.0167 | 0.0007887 |  | 4.65829847 |  | <https://docs.rs-online.com/4815/0900766b810cdcc0.pdf> |
| Panasonic - BSG | AM-5605 | 3.3 | 0.1154 | 0.00733894 |  | 5.18903275 |  | <https://docs.rs-online.com/8325/0900766b80d10cf2.pdf> |
| Panasonic - BSG | AM-5608 | 3.3 | 0.036 | 0.00248213 |  | 4.78621184 |  | <http://www.farnell.com/datasheets/87126.pdf> |
| Panasonic - BSG | AM-5610 | 3.3 | 0.0051 | 0.0005 |  | 3.366 |  | <https://docs.rs-online.com/8d09/0900766b80d10ce9.pdf> |
| Panasonic - BSG | AM-5613 | 3.3 | 0.0316 | 0.00220567 |  | 4.72781513 |  | <https://docs.rs-online.com/c5bd/0900766b810cdccf.pdf> |
| Panasonic - BSG | AM-5706 | 3.9 | 0.0459 | 0.0035 |  | 5.11457143 |  | <https://docs-asia.electrocomponents.com/webdocs/0d10/0900766b80d10cfb.pdf> |
| Panasonic - BSG | AM-5710 | 3.9 | 0.0326 | 0.0023051 |  | 5.51559585 |  | <http://www.farnell.com/datasheets/87128.pdf> |
| Panasonic - BSG | AM-5812 | 4.5 | 0.0198 | 0.0016933 |  | 5.2619146 |  | <https://docs.rs-online.com/cef8/0900766b810cdcf2.pdf> |
| Panasonic - BSG | AM-5813 | 4.5 | 0.025 | 0.00248024 |  | 4.53585137 |  | <https://docs.rs-online.com/559a/0900766b810cdcf7.pdf> |
| Panasonic - BSG | AM-5814 | 4.5 | 0.0386 | 0.00331151 |  | 5.24534125 |  | <https://docs.rs-online.com/5ed0/0900766b810cdd10.pdf> |
| Panasonic - BSG | AM-5815 | 4.5 | 0.0025 | 0.00033696 |  | 3.33867521 |  | <https://docs.rs-online.com/b981/0900766b810cdd26.pdf> |
| Panasonic - BSG | AM-5816 | 4.5 | 0.0065 | 0.00075756 |  | 3.86108031 |  | <https://panasonic.co.jp/ls/psam/en/products/pdf/Catalog_Amorton_ENG.pdf> |
| Panasonic - BSG | AM-5902 | 5 | 0.0608 | 0.005625 |  | 5.40444444 |  | <https://panasonic.co.jp/ls/psam/en/products/pdf/Catalog_Amorton_ENG.pdf> |
| Panasonic - BSG | AM-5904 | 5 | 0.0099 | 0.00132731 |  | 3.72934733 |  | <https://docs-apac.rs-online.com/webdocs/0d10/0900766b80d10ceb.pdf> |
| Panasonic - BSG | AM-5909 | 5 | 0.0222 | 0.00248213 |  | 4.47196561 |  | <https://docs.rs-online.com/5c4e/0900766b810cdd2f.pdf> |
| Panasonic - BSG | AM-5912 | 5 | 0.0153 | 0.00202488 |  | 3.77800166 |  | <https://docs.rs-online.com/a265/0900766b810cdd3d.pdf> |
| Panasonic - BSG | AM-5913 | 5 | 0.0301 | 0.00331151 |  | 4.54475451 |  | <http://www.farnell.com/datasheets/87130.pdf> |
| Panasonic - BSG | AM-5914 | 5 | 0.0231 | 0.00276051 |  | 4.18400948 |  | <https://docs.rs-online.com/6b14/0900766b810cdd4e.pdf> |
| Panasonic - BSG | AM-5E02 | 7.7 | 0.0232 | 0.004125 |  | 4.33066667 |  | <https://panasonic.co.jp/ls/psam/en/products/pdf/Catalog_Amorton_ENG.pdf> |
| Panasonic - BSG | AM-5S06 | 15.4 | 0.0114 | 0.00367275 |  | 4.78006943 |  | <https://panasonic.co.jp/ls/psam/en/products/pdf/Catalog_Amorton_ENG.pdf> |
| Panasonic - BSG | AM-7A03 | 5.5 | 0.227 | 0.02475 |  | 5.04444444 |  | <https://docs.rs-online.com/d908/0900766b80d10cea.pdf> |
| Panasonic - BSG | AM-7D08 | 7.2 | 0.172 | 0.02475 |  | 5.00363636 |  | <https://docs-asia.electrocomponents.com/webdocs/0d10/0900766b80d10cf0.pdf> |
| Panasonic - BSG | AM-7E04 | 7.7 | 0.104 | 0.0165 |  | 4.85333333 |  | <https://docs-apac.rs-online.com/webdocs/0d10/0900766b80d10cf9.pdf> |
| Panasonic - BSG | AM-7S03 | 15.4 | 0.07 | 0.02475 |  | 4.35555556 |  | <http://www.farnell.com/datasheets/87131.pdf> |
| Panasonic - BSG | AM-8703 | 3.9 | 0.0321 | 0.00227012 |  | 5.51468645 |  | <https://pdf1.alldatasheet.com/datasheet-pdf/view/251231/SANYO/AM-8703.html> |
| Panasonic - BSG | AM-8704 | 3.9 | 0.0238 | 0.00170156 |  | 5.45499424 |  | <https://pdf1.alldatasheet.com/datasheet-pdf/view/251232/SANYO/AM-8704.html> |
| Panasonic - BSG | AM-8706 | 3.9 | 0.0199 | 0.00149093 |  | 5.20547578 |  | <https://pdf1.alldatasheet.com/datasheet-pdf/view/251234/SANYO/AM-8706.html> |
| Panasonic - BSG | AT-7664 | 3 | 0.104 | 0.008176 |  | 3.81604697 |  | <https://docs-apac.rs-online.com/webdocs/0d10/0900766b80d10e9a.pdf> |
| Panasonic - BSG | AT-7665 | 3 | 0.0386 | 0.0032704 |  | 3.54085127 |  | <https://docs.rs-online.com/fd01/0900766b80d10e97.pdf> |
| Panasonic - BSG | AT-7666 | 3 | 0.343 | 0.024455 |  | 4.20772848 |  | <https://docs.rs-online.com/3ced/0900766b80d10e9e.pdf> |
| Panasonic - BSG | AT-7705 | 3.5 | 0.0333 | 0.003066 |  | 3.80136986 |  | <https://panasonic.co.jp/ls/psam/en/products/pdf/Catalog_Amorton_ENG.pdf> |
| Panasonic - BSG | AT-7802 | 4 | 0.0297 | 0.003066 |  | 3.87475538 |  | <https://panasonic.co.jp/ls/psam/en/products/pdf/Catalog_Amorton_ENG.pdf> |
| Panasonic - BSG | AT-7963 | 4.5 | 0.223 | 0.024455 |  | 4.10345533 |  | <https://docs.rs-online.com/028e/0900766b810cdd9b.pdf> |
| Panasonic - BSG | AT-7S63 | 15 | 0.134 | 0.049056 |  | 4.09735812 |  | <https://panasonic.co.jp/ls/psam/en/products/pdf/Catalog_Amorton_ENG.pdf> |
| Panasonic - BSG | AT-7S64 | 15 | 0.269 | 0.098112 |  | 4.11264677 |  | <https://panasonic.co.jp/ls/psam/en/products/pdf/Catalog_Amorton_ENG.pdf> |
| Seeed Technology Co., Ltd | SKU 114990057 | 2 | 0.25 | 0.01131 |  | 4.42086649 |  | <https://www.seeedstudio.com/2V-0-5W-Thin-film-Flexible-Solar-Panel-p-1857.html> |
| SparkFun Electronics | PRT-14795/SPT7.2-37 | 7.2 | 0.0105 | 0.0027 |  | 2.8 |  | <https://cdn.sparkfun.com/assets/2/1/9/f/9/SPT7.2-37_Solar_Panel_Spec_Sheet__2018_.pdf> |
| SparkFun Electronics | PRT-14797/PT15-300 | 15.4 | 0.2 | 0.087425 |  | 3.52301973 |  | <https://cdn.sparkfun.com/assets/3/2/1/2/4/PT15-300_Solar_Panel_Spec_Sheet__2018_.pdf> |
| SparkFun Electronics | PRT-14799/MPT4.8-75-KA | 4.8 | 0.05 | 0.00657 |  | 3.65296804 |  | <https://cdn.sparkfun.com/assets/8/7/d/7/d/MPT4.8-75-KA_Solar_Panel_Spec_Sheet__2018_.pdf> |
|  | | | | |  | |  |  |
|  |  |  |  |  | Average Efficiency | 4.4592767 |  |  |

**Table. S1.** Analysis of standard efficiency of commercial amorphous silicon panels using the average efficiency of +40 commercially available amorphous silicon solar panels produced by three different manufacturer and can be readily purchased through major electronic supplying companies.

**Experimental measurements and data**

Supplementary Figures. S1-S6 below show the measured short circuit current density (J_sc)_ and open circuit voltage (V_oc)_, as well as the power density derived by the J_sc_ and V_oc_ for a flat panel and with the addition of our 3D printed multi-element lenslet array (MELA), over latitudinal angles of 0°, 20°, 40°, 60°, 70°, 80°, respectively. These data were used to derive the power enhancement factors shown in Figure. 6.


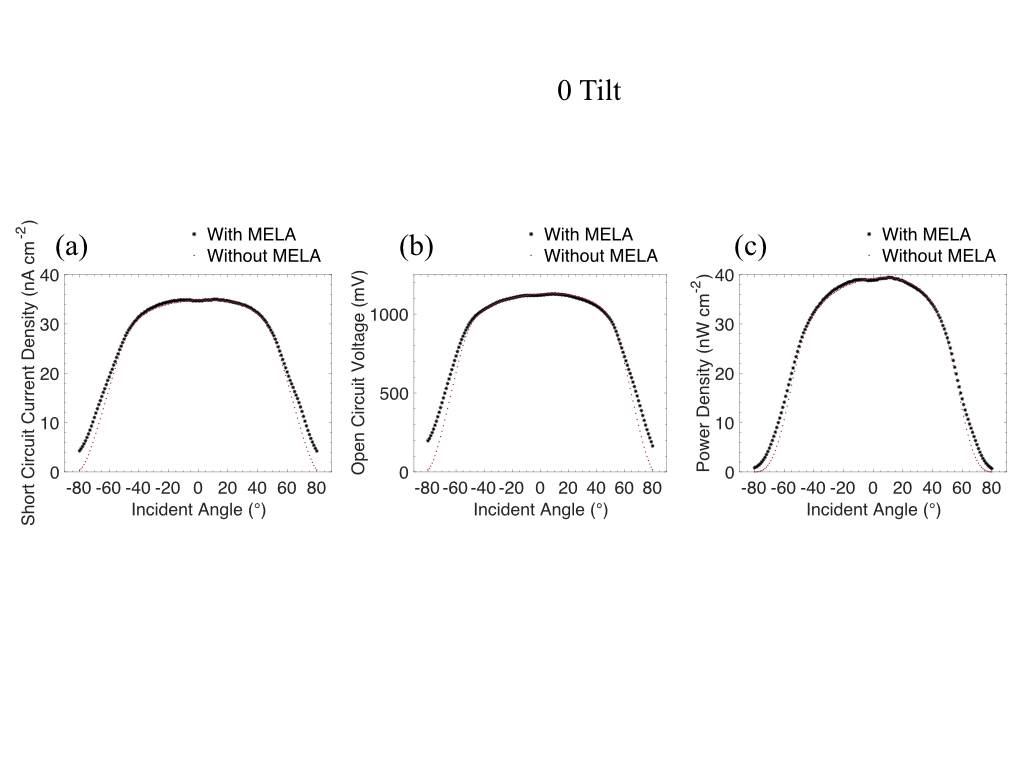


**Figure. S1.** Experimental testing at latitudinal angle of 0°. (a) Short circuit current density measurement through a flat solar panel (red) and with the addition of our 3D printed multi-element lenslet array (MELA) on-top of the same solar panel (black) over ±80° longitudinal incident angles, both under the same 0° latitude tilt illumination of a fiber-coupled stabilised broadband light source. The solar panel used here is an off-the-shelf amorphous silicon solar panel (Sanyo, AM-1815CA), which has a typical short circuit current density of 1.853 μA cm^-2^ per the manufacturer rating. (b) Open circuit voltage measurement across the flat panel and with the addition of our 3D printed optical element. (c) Power density produced by the flat panel and for the addition of our 3D printed MELA, which have been derived by the measurements taken in (a) and (b).


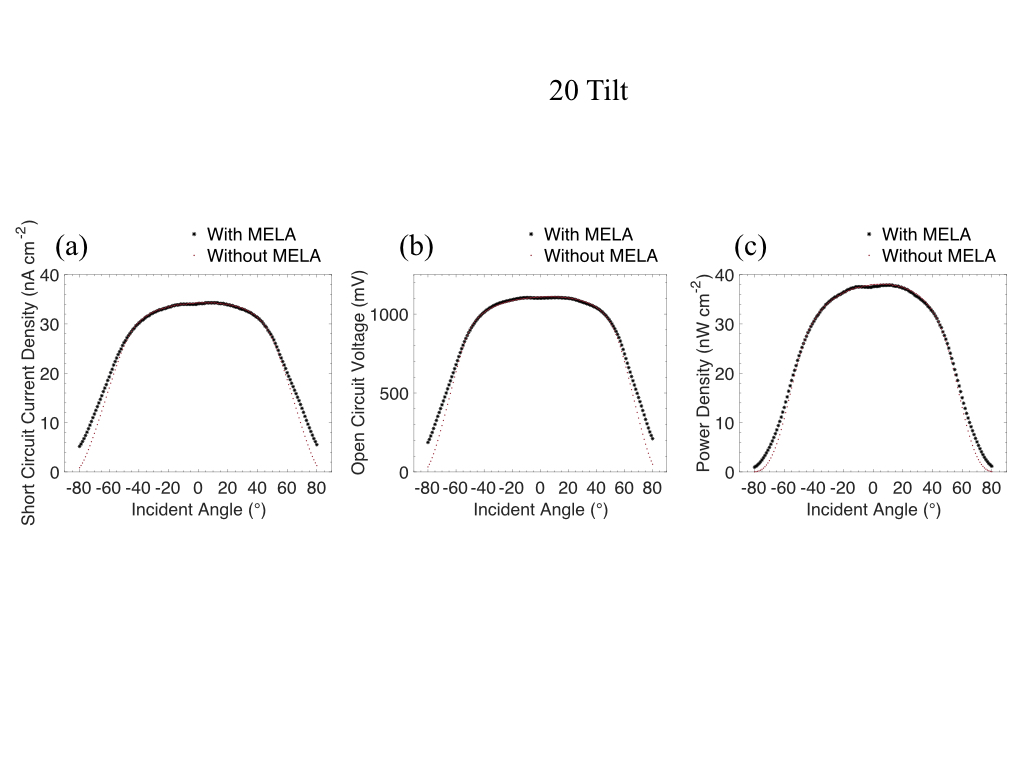


**Figure. S2.** Experimental testing at latitudinal angle of 20°. (a) Short circuit current density measurement through a flat solar panel (red) and MELA-integrated panel over ±80° longitudinal incident angles and a fixed 20° latitude tilt angle. (b) Open circuit voltage measurement across the flat panel and with the addition of our 3D printed MELA. (c) Power density produced by the flat panel and for the addition of our 3D printed MELA, which have been derived by the measurements taken in (a) and (b).


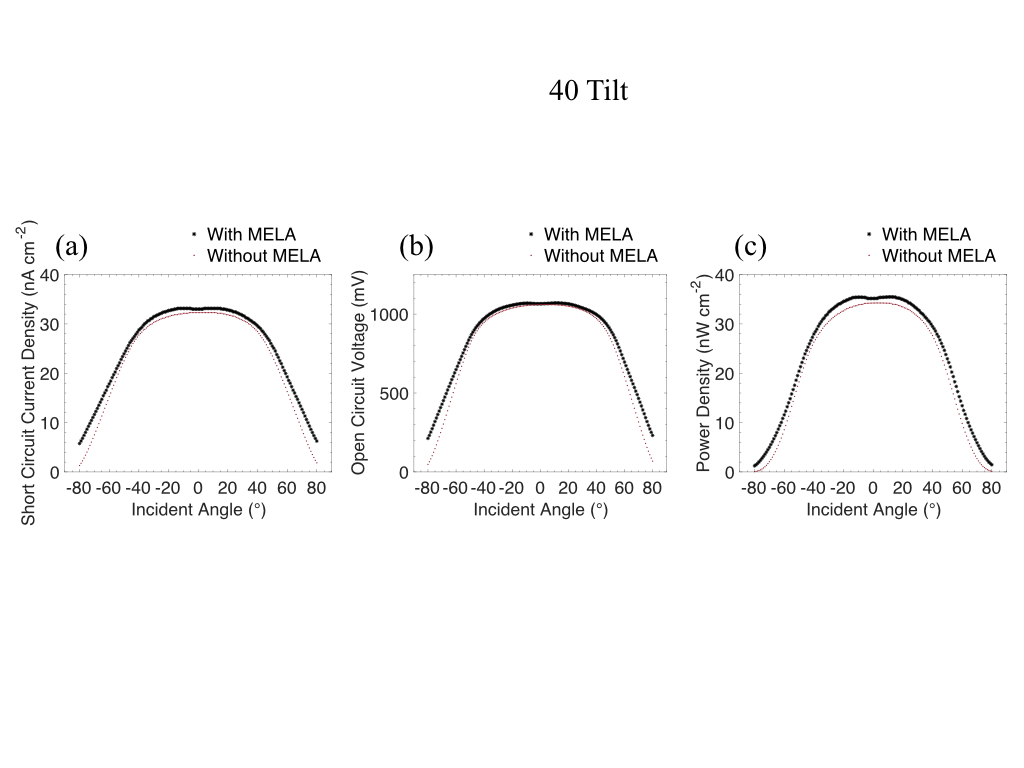


**Figure. S3.** Experimental testing at latitudinal angle of 40°. (a) Short circuit current density measurement through a flat solar panel (red) and MELA-integrated panel over ±80° longitudinal incident angles and a fixed 40° latitude tilt angle. (b) Open circuit voltage measurement across the flat panel and with the addition of our 3D printed MELA. (c) Power density produced by the flat panel and for the addition of our 3D printed MELA, which have been derived by the measurements taken in (a) and (b).


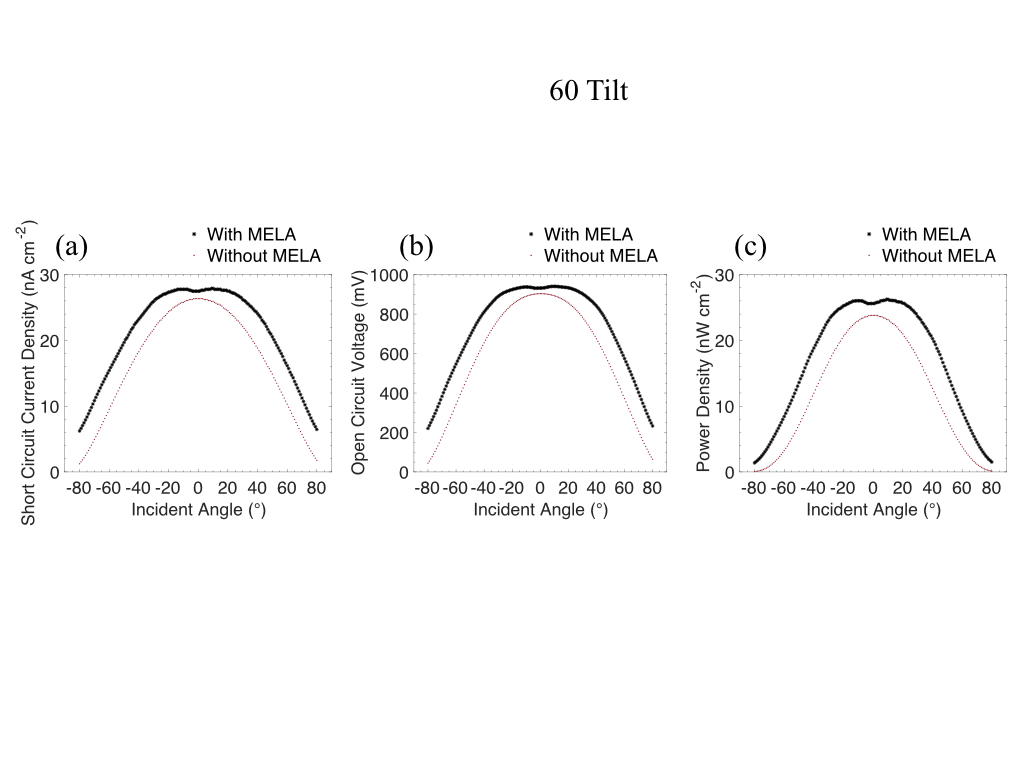


**Figure. S4.** Experimental testing at latitudinal angle of 60°. (a) Short circuit current density measurement through a flat solar panel (red) and MELA-integrated panel over ±80° longitudinal incident angles and a fixed 60° latitude tilt angle. (b) Open circuit voltage measurement across the flat panel and with the addition of our 3D printed MELA. (c) Power density produced by the flat panel and for the addition of our 3D printed MELA, which have been derived by the measurements taken in (a) and (b).


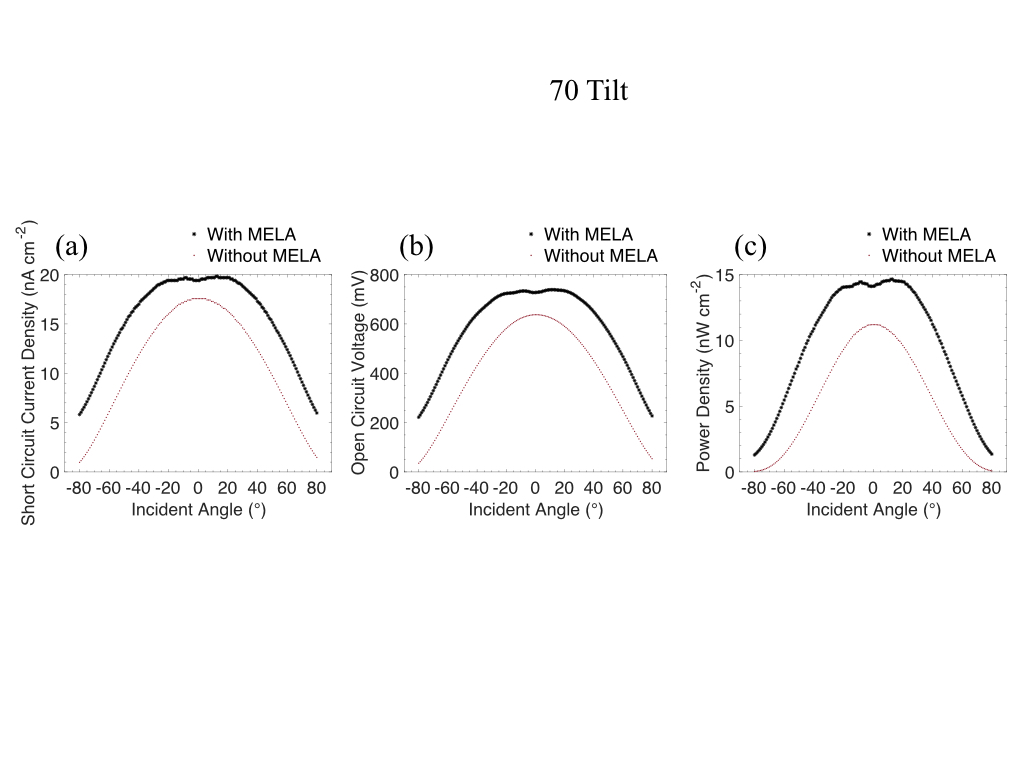


**Figure. S5.** Experimental testing at latitudinal angle of 70°. (a) Short circuit current density measurement through a flat solar panel (red) and MELA-integrated panel over ±80° longitudinal incident angles and a fixed 70° latitude tilt angle. (b) Open circuit voltage measurement across the flat panel and with the addition of our 3D printed MELA. (c) Power density produced by the flat panel and for the addition of our 3D printed MELA, which have been derived by the measurements taken in (a) and (b).


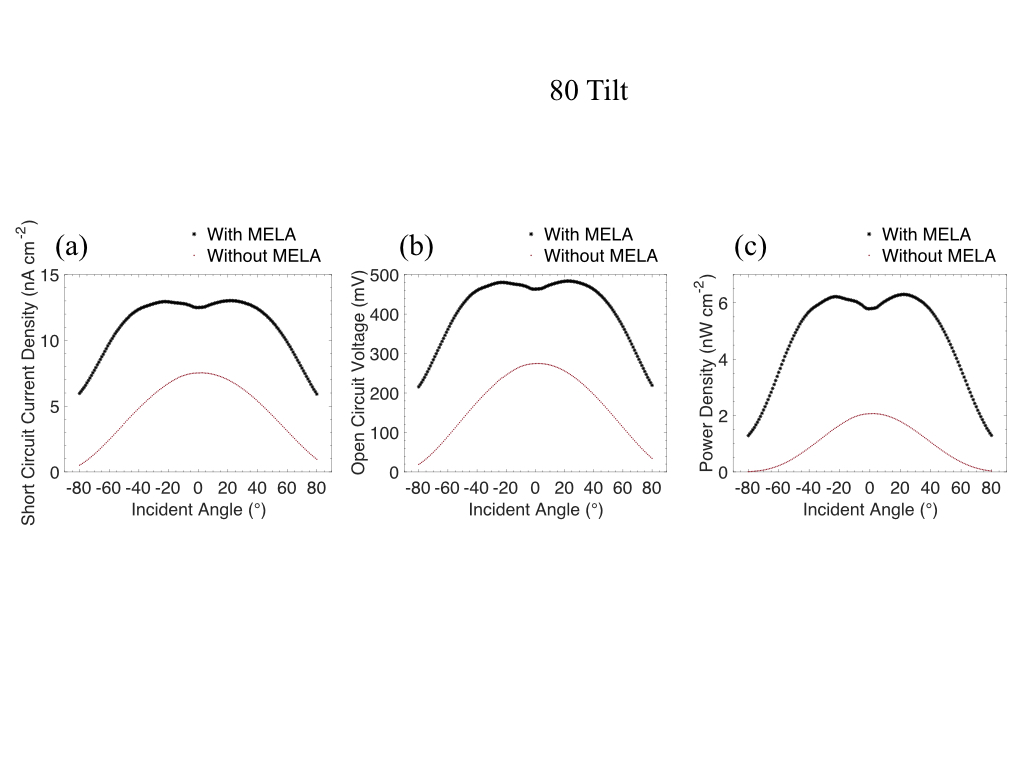


**Figure. S6.** Experimental testing at latitudinal angle of 80°. (a) Short circuit current density measurement through a flat solar panel (red) and MELA-integrated panel over ±80° longitudinal incident angles and a fixed 80° latitude tilt angle. (b) Open circuit voltage measurement across the flat panel and with the addition of our 3D printed MELA. (c) Power density produced by the flat panel and for the addition of our 3D printed MELA, which have been derived by the measurements taken in (a) and (b).

**Loss characterisation**

**

**Figure. S7.** System evaluation with Neutral Density (ND) filters. Power produced by the solar panel at different optical density from 0.1 to 1 with a step of 0.1.

Neutral Density (ND) filters were used to evaluate the performance of the amorphous silicon solar panel under different optical intensities. The optical density (OD) was varied from 0.1 to 1 in steps of 0.1 to directly measure the variance in produced power with respect to optical intensity variations for our specific solar panel (AM-1815CA, Sanyo, Tokyo, Japan), plotted in Supplementary Figure. S7. The most conservative percentage loss in the produced power due to corresponding reduction in optical intensity was used to interpolate the power gain for a 13.41% optical loss, which is the difference between our 3D printed element and anti-reflective coated PMMA injection molded design.
